# Outcomes of Bebtelovimab Therapy in Patients With Solid Organ Transplantation With Mild and Moderate COVID-19

**DOI:** 10.7759/cureus.38867

**Published:** 2023-05-11

**Authors:** Rohan Kapur, Kenji Okumura, Nicholas Feola, Marina Keller, Abhay Dhand

**Affiliations:** 1 Internal Medicine, Westchester Medical Center, Valhalla, USA; 2 Surgery, Westchester Medical Center/New York Medical College, Valhalla, USA; 3 Pharmacy, Westchester Medical Center, Valhalla, USA; 4 Infectious Diseases, Westchester Medical Center, Valhalla, USA; 5 Transplant Infectious Disease, Medicine, and Surgery, Westchester Medical Center/New York Medical College, Valhalla, USA

**Keywords:** monoclonal antibody treatment for covid-19, covid-19, monoclonal antibody, kidney transplant, liver transplant, solid organ transplant, bebtelovimab

## Abstract

Solid organ transplant recipients (SOTRs) are at greater risk of poorer outcomes from coronavirus disease 2019 (COVID-19) as compared to the general population. Because of significant drug-drug interactions between nirmatrelvir-ritonavir and immunosuppressive agents as well as logistical challenges of outpatient administration of remdesivir, anti-severe acute respiratory syndrome coronavirus-2 (SARS-CoV-2) monoclonal antibodies (mAbs) had been the mainstay of outpatient treatment of COVID-19 among SOTRs, with bamlanivimab, casirivimab-imdevimab, and sotrovimab having been previously granted emergency use authorization by the Food and Drug Administration (FDA). The challenge with the ongoing use of these monoclonal antibodies is the loss of efficacy against emerging variants of SARS-CoV-2. Bebtelovimab, which retained efficacy against early subvariants of Omicron, was granted emergency use authorization by the Food and Drug Administration when Omicron BA.4 and BA.5 became the predominant variants in the United States. However, the study based on which bebtelovimab was authorized by the FDA did not include SOTRs. The only available safety and efficacy data on these patients are from retrospective studies. In our retrospective analysis of 62 SOTRs who received bebtelovimab infusion between May 11, 2022, and October 11, 2022, 28 had a kidney transplant, 18 had a liver transplant, 10 had a heart transplant, and six had multi-organ transplants (liver/kidney: 4, heart/kidney: 2). None of the patients reported infusion-associated adverse reaction. Only one (1.6%) patient developed progression of COVID-19, requiring subsequent treatment with remdesivir, steroids, and oxygen supplementation. The rate of need for intensive care and death from COVID-19 during the 30-day follow-up period was 0%.

## Introduction

Solid organ transplant recipients (SOTRs) have a much higher incidence of coronavirus disease 2019 (COVID-19) (approximately 15 times higher) compared to the general population and are also at a higher risk for worse outcomes [1,2]. In a study of 1,013 SOTRs across Europe, the 28-day mortality risk was approximately 20%-30% [3], in contrast to 0.8%-2% in the general population [4]. However, it remains unclear whether SOTRs have a higher COVID-19-attributable mortality rate than the general population, which is matched for demographic and comorbidity factors. Besides immunosuppression (IS), SOTRs frequently have other risk factors of progression to severe COVID-19, including increased body mass index (BMI) and underlying organ dysfunction including chronic lung disease, kidney dysfunction, diabetes, and hypertension [5]. SOTRs have variable and unpredictable vaccine efficacy, which can be associated with age, cumulative IS, time from transplant, and willingness to receive multiple follow-up doses of COVID vaccines [6]. Early administration of anti-severe acute respiratory syndrome coronavirus 2 (SARS-CoV-2) monoclonal antibody (mAb) in these patients has emerged to be a safe outpatient therapy, reducing the risk of hospitalization, admission to intensive care unit (ICU), and death [7].

Casirivimab-imdevimab, bamlanivimab, and sotrovimab have previously been approved for the treatment of mild to moderate COVID-19 in high-risk patients, including SOTRs [6]. However, the use of mAbs is limited by the rapidly evolving variants of SARS-CoV-2 during the pandemic. Therefore, many previously effective mAbs can no longer be used [8]. Bebtelovimab is a human immunoglobulin-G1-lambda (IgG1λ) mAb, created by genetic engineering, that aims at a conserved epitope on the receptor-binding domain found on the spike glycoprotein of the SARS-CoV-2 virus [9]. In February 2022, the Food and Drug Administration (FDA) granted emergency use authorization for this drug for the treatment of mild to moderate COVID-19 [9]. In phase 2 BLAZE-4 trial, bebtelovimab, when used alone or in conjunction with bamlanivimab-etesevimab, led to greater viral clearance and faster resolution of symptoms compared with placebo [10]. Unlike other mAbs previously in use, bebtelovimab was found to retain neutralizing in vitro activity against predominant Omicron subvariants, including BA.1.1, BA.2, and BA.4.5 [8]. Like in studies with other mAbs, SOTRs were not included in the BLAZE-4 trial based on which bebtelovimab obtained FDA emergency use authorization. The evidence for the safety and efficacy of this medication in this high-risk population has only come from retrospective studies. We aim to add to the literature by performing a retrospective study on SOTRs who received bebtelovimab for the treatment of mild to moderate COVID-19 at our medical center.

## Materials and methods

This study was reviewed by the New York Medical College Institutional Review Board and granted an exempt status. This is a retrospective study conducted at a single transplant center in New York and included all adult SOTRs who received bebtelovimab for the treatment of mild to moderate COVID-19 between May 11, 2022, and October 11, 2022. During this time, bebtelovimab had emergency use authorization from the FDA for use in this patient population. Patients who were requiring oxygen support qualified as having severe COVID-19 and were not treated with this medication. Bebtelovimab was given as a single dose of 175 mg as an intravenous push. The diagnosis of COVID-19 was confirmed by a polymerase chain reaction (PCR) test performed on a nasopharyngeal swab from the patient. The research team collected data on patient demographics, transplant characteristics, COVID-19 vaccination status, prior prophylaxis with tixagevimab-cilgavimab, comorbidities, presenting symptoms, duration of symptoms prior to treatment, oxygen saturation at the time of treatment, and medications used for immunosuppression.

The outcomes evaluated in the study were the safety of the infusion, progression of COVID-19 symptoms, COVID-19-associated hospitalizations, ICU admissions, and mortality within 30 days of COVID-19 diagnosis. Data was collected retrospectively by examining the electronic health records of the patients.

## Results

**Figure 1:**
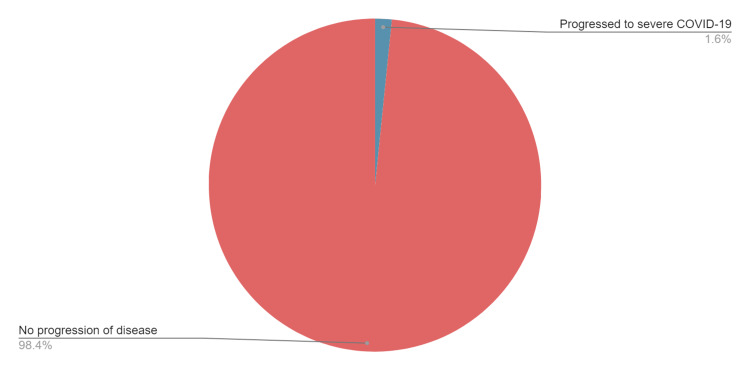
Progression to severe COVID-19 in transplant recipients with mild to moderate COVID-19 treated with bebtelovimab COVID-19: coronavirus disease 2019

During the study period, Omicron BA.4 and BA.5 subvariants of SARS-CoV-2 caused most of the COVID-19 cases in New York. Bebtelovimab was given to 62 adult SOTRs for the treatment of mild-moderate COVID-19 after meeting the FDA emergency use authorization criteria. The average duration of symptoms prior to treatment was 2.8 days (range: 1-7 days), and the most common presenting symptom was cough, seen in 33 (53%) patients. The setting of infusion was outpatient in 76% of SOTRs and inpatient in 24% SOTRs with non-COVID-19 hospitalizations. Types of transplants were kidney (45%), liver (29%), heart (16%), and multi-organ transplant (10%). Patients were on average 5.9 years away from transplant (range: 0.1-24.2 years). The immunity status of patients included any dose of COVID-19 vaccination in 56 (90%), unvaccinated in six (10%), and having received tixagevimab-cilgavimab as pre-exposure prophylaxis in 11 (18%) of the patients. Patients were monitored for one hour after the administration of the drug, and no infusion-associated adverse reactions were noted. All patients had a follow-up of at least 30 days after the administration of bebtelovimab. Out of 62 patients who received the drug, only one (1.6%) patient developed worsening COVID-19 symptoms, subsequently requiring treatment with remdesivir, steroids, and oxygen supplementation (Figure 1). This was a 61-year-old male patient with a dual liver-kidney transplant; he also had other comorbidities such as diabetes and hypertension. None of the patients died, and none required a stay in the intensive care unit due to worsening of COVID-19. These results are summarized in Table 1.

**Table 1:** Patient characteristics and outcomes of bebtelovimab in solid organ transplant recipients with mild to moderate COVID-19 COVID-19: coronavirus disease 2019, BMI: body mass index

Characteristic	Result
Age, mean (range), years	54.6 (19-79)
Female sex, number (%)	29 (47)
Medication administration, number (%)	
Outpatient	47 (76)
Inpatient	15 (24)
Type of transplant, number (%)	
Kidney	28 (45)
Liver	18 (29)
Heart	10 (16)
Liver-kidney	4 (6)
Heart-kidney	2 (3)
BMI, mean (range), kg/m2	28.6 (18.2-58.2)
Comorbidities, number (%)	
Diabetes mellitus	23 (37)
Chronic kidney disease	35 (56)
Heart disease	49 (79)
Duration of symptoms prior to administration, mean (range), days	2.8 (1-7)
Predominant presenting symptoms, number (%)	
Fever/chills	23 (37)
Cough	33 (53)
Nasal congestion	30 (48)
Malaise	21 (34)
Sore throat	13 (21)
Oxygen saturation on room air, mean (range), %	97.7 (94-100)
Time from transplant, mean (range), years	5.9 (0.1-24.2)
Adverse drug reaction, number (%)	0 (0)
Progression of COVID-19 symptoms, number (%)	1 (1.6)
COVID-19 vaccination status, number (%)	
Vaccinated (any dose)	56 (90)
Unvaccinated	6 (10)
Immunosuppression, number (%)	
Tacrolimus	51 (82)
Mycophenolate	35 (56)
Cyclosporine	6 (10)
Sirolimus	3 (5)
Prior tixagevimab-cilgavimab administration, number (%), mg	
300 mg/300 mg	8 (13)
150 mg/150 mg	3 (5)
Time since tixagevimab-cilgavimab administration, mean (range), days	115 (22-201)

## Discussion

Anti-SARS-CoV-2 monoclonal antibodies were the mainstay of treatment for mild-moderate COVID-19 in severely immunocompromised patients, including SOTRs, across various periods of the COVID-19 pandemic in the United States. Since most transplant recipients were excluded from the clinical trials that led to the approval of these mAbs, the data regarding their safety and efficacy in these high-risk patients is limited.

Previously, Yetmar et al. [11] showed that 92 SOTRs who received bebtelovimab in a large network-wide study had similar 30-day outcomes to other 269 SOTRs who received treatment with sotrovimab. In the bebtelovimab cohort, no immediate adverse events were noted, the rate of hospitalization due to worsening COVID-19 was 3.3%, none of the patients required ICU care, and none died. In the experience by Shertel et al. [12], among 25 SOTRs who received bebtelovimab on an average of three days from onset of symptoms, only one (4%) experienced progression of symptoms requiring hospitalization, none required ICU care, and there were no deaths noted during the 30-day follow-up period. McCreary et al. [13] showed that outpatient treatment with bebtelovimab in immunocompromised patients was associated with a lower risk of hospitalization or death (adjusted odds ratio: 0.50). In a nurse-driven monoclonal antibody program by Cochran et al. [14], among 145 SOTRs who received bebtelovimab, 18 (12.4%) required hospitalization, one (0.7%) required mechanical ventilation, and one (0.7%) died. In a retrospective cohort study by Razonable et al. [15], among 2,833 high-risk patients treated with bebtelovimab, the rate of progression to severe disease was 1.4%, which was not significantly different from that for nirmatrelvir-ritonavir treatment (1.2%) given to 774 patients during the same study period. In this study, older patients with higher comorbidities preferentially received bebtelovimab [15]. Our results add to the prior limited studies in SOTRs in proving the efficacy of bebtelovimab in reducing the risk of progression of COVID-19, hospitalization, need for intensive care, or death.

Bebtelovimab followed other mAbs such as bamlanivimab, bamlanivimab-etesevimab, casirivimab-imdevimab, and sotrovimab, which were successfully utilized to treat mild-moderate COVID-19 in previous eras of the pandemic [6]. Eventually, all these mAbs lost their efficacy after the emergence of various variants of SARS-CoV-2, thus highlighting the strengths and potential weaknesses of passive immunity in the treatment of various infectious etiologies [6]. These therapies also show effectiveness when given early in the disease, which requires a dedicated pathway for early diagnosis and a mechanism in place for early infusion of mAbs. This process can be at risk of various regional and socioeconomic disparities, as this medication can be prohibitively expensive without insurance coverage [16]. Furthermore, passive immunity is short lasting. Therefore, the impact of these interventions is not durable. However, in severely immunocompromised patients, including SOTRs, where vaccine efficacy is unreliable and often poor, mAbs may still offer an efficacious and safe option for the treatment of COVID-19 in a strain-specific manner [6]. Because the newer variants of Omicron were not susceptible to bebtelovimab, emergency use authorization was reversed by the FDA in November 2022 [17].

The limitations of our study include its retrospective nature with no control group and a small sample size.

## Conclusions

In our single-center retrospective study, bebtelovimab was a safe and efficacious treatment option for SOTRs with mild-moderate COVID-19 during the Omicron epoch in the United States. The use of bebtelovimab in high-risk adult SOTRs was associated with decreased risk of progression of symptoms, need for hospitalization, need for intensive care, and death associated with COVID-19. This adds to the real-world evidence regarding its safety and efficacy in SOTRs. Also, this data adds to the existing evidence regarding the use of passive immunity using polyclonal or targeted mAbs for the management of various infectious etiologies including COVID-19. Due to the development of further variants of Omicron, bebtelovimab lost its efficacy against the newer variants, and the emergency use authorization was discontinued by the FDA.
